# Role of Digital Media in Promoting Oral Health: A Systematic Review

**DOI:** 10.7759/cureus.28893

**Published:** 2022-09-07

**Authors:** Shristy Sharma, Vikrant Mohanty, Aswini Y Balappanavar, Puneet Chahar, Kavita Rijhwani

**Affiliations:** 1 Public Health Dentistry, Maulana Azad Institute of Dental Sciences, New Delhi, IND

**Keywords:** pediatric preventive dentistry, dental education, dental caries, oral health care, promoting oral health, oral health, digital media

## Abstract

Oral disease has affected almost half of the world’s population, causing an enormous economic burden. To overcome this huge problem, oral health promotion is one of the most cost-effective methods. Digital media can play a pivotal role in achieving the goal of reducing this burden by providing a wider platform to reach out to the population even in the areas of deficit oral health care service.

The aim of this systematic review is to assess the effectiveness of digital media in oral health promotion.

The combinations of terms in the following two broad categories were used to search the literature on PubMed, Cochrane Library articles, WHO guidelines on oral health promotion through digital media, and Google Scholar: Intervention (digital media, Mobile phones, Text messages, social media, Cell phones, MHealth application, Telemedicine, Television, Videos) and Outcome (Oral Health education, Oral health, Oral health promotion, Oral health literacy, Oral health knowledge, Oral health attitude, Oral health practice, Oral hygiene improvement). The review was conducted in two phases, using the standardized checklist applicable to studies. Initially, abstracts were retrieved, followed by the assessment of the full papers against the review criteria.

Among the selected studies, digital interventions helped in providing continuity of care and services in seven studies, eight addressed the increase in knowledge, attitude, and practice, and all the studies analyzed oral health needs.

The findings concluded that digital media-based interventions can enhance oral health literacy and help in tackling this problem among different age groups.

## Introduction and background

Oral diseases are among the most prevalent diseases globally and have serious health and economic burdens [1]. It is estimated that oral diseases affect nearly 3.5 billion people [2]. Untreated dental caries (tooth decay) in permanent teeth is the most common health condition according to the Global Burden of Disease 2017 [3]. They have a great impact on reducing the quality of life [1]. Poor oral health is one of the risk factors for many diseases like coronary artery disease, cancer, and diabetes, but it is neglected.

High-income countries being equipped with high technology and specialized treatment approach still failed to address the oral health inequality. While on the other hand, in middle-low-income countries, dentistry is often unavailable, unaffordable, and inappropriate for the majority of these populations particularly the rural poor [4]. According to a survey, oral health care services access ranges from 35% in low-income countries to 60% in lower-middle-income countries, 75% in upper-middle-income countries, and 82% in high-income countries [5]. Moreover, even in high-income settings, dental treatment is costly, averaging 5% of total health expenditure and 20% of out-of-pocket health expenditure [6]. Oral diseases are usually not considered a priority in public health policies [7], often ignored in primary healthcare and nursing in clinical and community settings [8], along with a lack of oral disease surveillance on the global stage [9]. One of the main factors for this negligence could be that poor oral health affects morbidity rather than mortality [10]. Owing to these factors, oral diseases pose a major health burden for many countries and affect people throughout their lifetime. Overcoming such a huge burden of prevention at the community or population-based level is the most cost-effective approach, which has led to a great impact on oral disease prevalence. Many different approaches to preventing dental diseases exist, and the most cost-effective method is health promotion and education [11].

Digital media are means of interactions among people in which individuals create, share, or exchange information and ideas in virtual (online or cloud-based) communities and networks [12]. Three-quarters of US adults use social media; of these, three-quarters engage at least once daily [13] and nearly 50% report that information found via social media affects the way they deal with their health. In China, more than 740 million individuals (> 50% of the population) have social media accounts with which they daily engage [14], and more than 70% of WeChat’s (a Chinese messaging, social media, and mobile payment app) 570 million users report it to be their primary source of health education [15]. As digital media access continues to expand, it will increasingly serve as a rich health resource in environments that lack health expertise. They are an alternative platform that can aid in reaching out to the wider population where oral health delivery services are also limited. Interactive digital interventions have been shown to increase knowledge of oral health and modify oral health behavior, which is one of the prime approaches to reducing the oral health burden.

We need to encourage evidence-based MHealth interventions to ensure patients' effective engagement and positive change in their behavior modifications toward oral health practices. Though there have been lots of digital media-based oral health interventions, there is still a systematic approach to address the effectiveness of digital media to reach out to a varied population base is required. So, the aim of this review is to access the effectiveness of digital media in oral health promotion and hence address the concern of trimming down the oral health disease burden.

## Review

Methodology 

Literature Search

**Figure 1:**
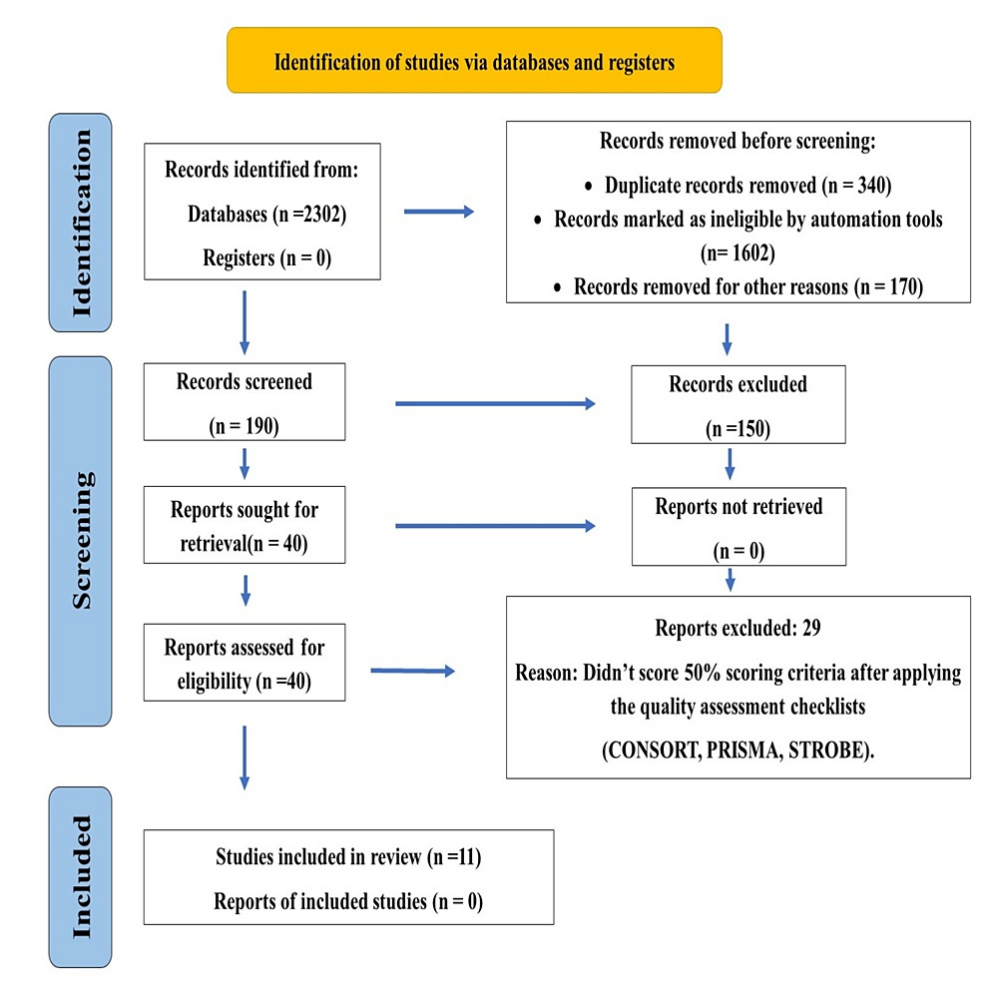
Study Flowchart

This systematic review followed the Preferred Reporting Items for Systematic Reviews and Meta-Analyses (PRISMA) guidelines. Two researchers built up a strategy that incorporated a combination of keywords and their synonyms. A literature search was done between June 2019 till October 2019 to identify the articles that provided the evaluation of the oral health promotion interventions through digital media platforms. Databases like PubMed, Google Scholar, and Cochrane Library were identified and searched for relevant articles. Due to changeable definitions, a wider range of search terms was used. A combination of terms in the following two broad categories was used to gather a range of literature for this review: Intervention (digital media, eHealth, MHealth, mobile phones, text messages, social media, cell phones, MHealth application, telemedicine, television, videos), Outcome (oral health education, oral health, oral health promotion, oral health literacy, oral health knowledge, oral health attitude, oral health practice, oral hygiene improvement). Where possible, PubMed MeSH terms were used with similar terms and phrases to broaden the search strategies. The literature search strategy and studies included in this systematic review are shown in Figure 1.

Inclusion and Exclusion Criteria

This review considered randomized controlled trials (RCTs), quasi-experimental trails, non-randomized trials, and systematic review study designs in which digital media is used in any form to enhance oral health literacy and address oral health needs. The full-text articles from 2010 to 2019 focus on participants of any age who have interacted with the oral health care system or participated in oral health management using digital technologies or interventions and compared the same with the traditional method (counseling on a face-to-face basis or through pamphlets/ booklets etc.) are included for the study. Editorials, conference papers, and interventions to improve overall health were excluded.

The articles were selected by three authors in two phases. The review was conducted in two phases: initially, abstracts were retrieved and followed by the retrieval and assessment of full papers against the review criteria. The inclusion and exclusion criteria were applied and 132 studies were chosen. The reporting quality of the selected articles was assessed using the CONSORT and PRISMA checklist. After applying the relevant checklist and considering a 50% score as the criterion for selecting the study, finally, 12 studies (7 randomized clinical trials, 1 quasi-experimental study, 1 non-randomized trial study, and 3 systematic reviews) were shortlisted for this systematic review. The 25% criterion was not used to maintain the quality of the review, and a 75% score may decrease the number of selected studies for the systematic analysis.

Data extraction from the final articles was done using a data extraction form adopted from the Cochrane Collaboration for RCT and non-RCT study design [16]. Furthermore, the outcomes of the research papers were reviewed under three main components, which were an increase in knowledge, attitude, and practice, an increase in continuity of care and services, and addressing oral health needs.

Results

This systematic review was done to assess the effectiveness of digital media to promote oral health and incorporate oral health into Universal Health Coverage. The studies included in this review consist of seven randomized clinical trials, one quasi-experimental trial, one non-randomized trial, and three systematic reviews.

Population of Interest

These studies were conducted in Iran [17-19], India [20,21], Italy [22], Netherland [23], Sydney [24], London [25], Brazil [26], Nigeria [27], and Syria [28]. Twenty-five percent (25%) of studies had considered primary school children, 25% considered adolescents, 8% study considered mothers of one to two-year-old children, 8% of studies considered 18-50-year-old adults, and 8% of studies did not consider any age bar. Among the systematic reviews, one had considered 15 articles with a sample size of 1402, and one had studied 58 research papers with a sample size of 17538 populations with no age limit specifically mentioned. The systematic review conducted by Bassi et al. (2018), considered 125 primary research and 193 secondary research papers.

Quality Assessment of the Selected Study

**Figure 2:**
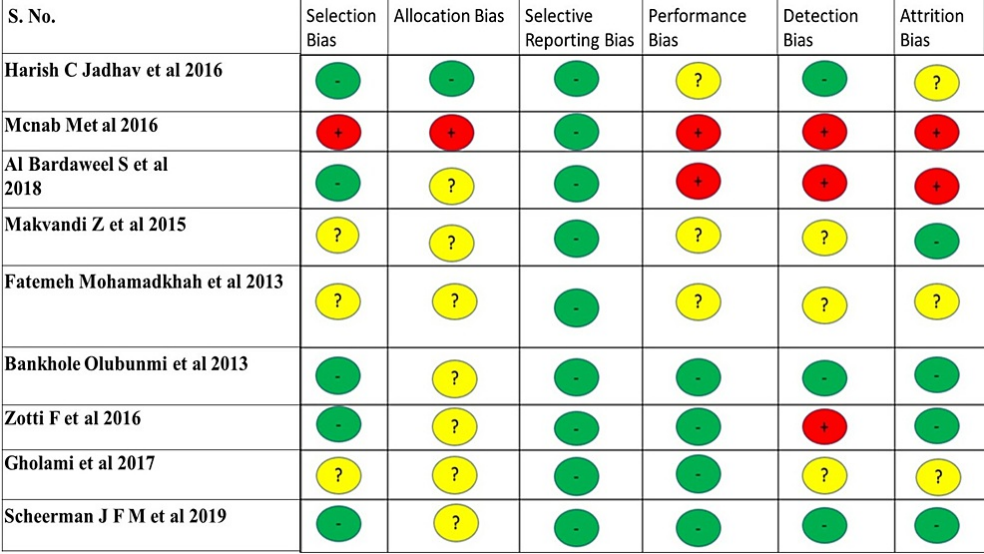
Cochrane Risk of Bias Tool '+' High Risk of Bias '-' Low Risk of Bias '?' Unclear Risk of Bias Harish C Jadhav et al. 2016 [21], Mcnab M et al. 2016 [24], Al Bardaweel S et al. 2018 [28], Makvandi Z et al. 2015 [17], Fatemeh Mohamadkhah et al. 2013 [19], Bankhole Olubunmi et al. 2013 [27], Zotti F et al. 2016 [22], Gholami et al. 2017 [18], Scheerman JFM et al. 2019 [23]

The quality assessment of the primary research is done using the Quality Criteria Checklist: Primary Research (Table 1) [29]. The quality of the systematic reviews was assessed using the AMSTAR 2 checklist (Table 2) [30]. The Cochrane risk of bias assessment tool was used to assess the risk of biases in randomized controlled trials (Figure 2) [31].

**Table 1:** Quality Criteria Checklist: Primary Research MINUS/NEGATIVE (-): If most (six or more) of the answers to the above validity questions are “No,” the report should be designated with a minus (-) symbol on the Evidence worksheet. NEUTRAL (∅): If the answers to validity criteria questions 2, 3, 6, and 7 do not indicate that the study is exceptionally strong, the report should be designated with a neutral (∅) symbol on the Evidence worksheet. PLUS/POSITIVE (+): If most of the answers to the above validity questions are “Yes” (including criteria 2, 3, 6, 7, and at least one additional “Yes”), the report should be designated with a plus symbol (+) on the Evidence worksheet.

S.No.	Reference Study	Quality Assessment
1.	Scheerman JFM, et al 2019 [23]	Positive
2.	Al Bardaweel S, et al 2018 [28]	Positive
3.	Gholami et al 2017 [18]	Neutral
4.	Zotti F et al 2016 [22]	Neutral
5.	Makvandi Z et al 2015 [17]	Neutral
6.	Harish C Jadhav et al 2015 [21]	Neutral
7.	Bankhole Olubunmi, 2013 [27]	Neutral
8.	Fatemeh Mohamadkhah et al 2013 [19]	Neutral
9.	Mcnab M, Skapetis et al 2016 [24]	Negative

**Table 2:** Amstar 2 Checklist High • No or one non-critical weakness Moderate • More than one non-critical weakness Low • One critical flaw with or without non-critical weaknesses Critically low • More than one critical flaw with or without non-critical weaknesses

S. No.	Reference Study	Rating Overall Confidence in the Results of the Review
1.	Toniazzo MP et al. 2019 [26]	High
2.	Caroline Free et a.l 2015 [25]	High

Intervention Design

To review the role of digital media in the promotion of oral health and increasing oral health literacy levels worldwide, three components were studied in each article. The components are an increase in knowledge, attitude, and practice, an increase in continuity of care and services, and addressing oral health needs. The digital media used in various interventions were 41.6% educational videos, 8% mobile applications (white teeth), 16% educational and reminder SMSs, and 8% Whatsapp chat rooms. The review articles considered SMS, mobile applications like Dell Axim×5 (Dell, Austin, Texas), Casio PB-750 personal digital assistant (Shibuya, Tokyo), educational videos for motivation, and maintaining a healthy lifestyle. The interventional study duration with follow-up ranges from 1 month (11%), 1.5 months (11%), 3 months (55%), 6 months (11%), and 1 year (11%) with a mean duration of 3.9 months. The follow-up retention rate of the interventional study was ranging from 54.1% to 100% with a mean of 87.8. Two interventional studies didn’t mention the retention rates during the follow-ups. The outcomes of the research papers were studied under three main components, which were an increase in knowledge, attitude, and practice, an increase in continuity of care and services, and addressing oral health needs (Tables 3, 4). All studies addressed the need for oral health and the use of digitalization in creating awareness of oral health.

**Table 3:** Increase in Knowledge, Attitude, and Practice (KAP)

S. No	Year	Reference Study	Result/Increase KAP
1	2019	Scheerman JFM et al. [23]	20.08-22.50%
2	2017	Gholami et al. [18]	0.29-0.61
3	2016	McNab M et al. [24]	57.5-78.25
4	2015	Makvandi Z et al. [17]	[K- 4.80-6.20], [A- 77.62-95], [P- 58.29-83.35]
5	2013	Mohamadkhah F et al. [19]	[K- 1.93-2], [A- 1.86-2.05], [P- 2.02-2.07]

**Table 4:** Increase in Continuity of Care and Services

S. No	Year	Reference Study
1.	2019	Toniazzo MP et al. [26]
2.	2018	Abhinav Bassi et al. [20]
3.	2016	McNab M et al. [24]
4.	2015	Harish C Jadhav et al. [21]
5.	2015	Makvandi Z et al. [17]
6.	2015	Free C et al. [25]

Outcome Measures

All the studies notified the importance of oral health and how we can use digital media and software to enhance the oral health literacy level and reduce the burden of dental diseases by preventing oral disease in the future. The increase in knowledge, attitude, and practice is shown by five studies [18,19,23,24] selected for this review. These studies show an increase in the mean score of knowledge (K; 4.80 to 6.68), attitude (A; 77.65 to 95.0), perceived behavioral control (58.29 to 83.35), and intention to perform that changed the behavior (4.09 to 4.72) [17], K 1.93 to 2, A 1.86 to 2.05, practice (P) 2.07 to 2.02 [19], KAP 57.5% to 78.2% [24], knowledge score regarding periodontal health increased by 0.29 to 0.61 [18], improving oral hygiene behavior among the intervention group by 20.08 % to 22.50% [23]. Zotti F et al. 2016 [22] showed that the plaque index score, when measured at the end of treatment, was less among the study group (0.41 to 1.06) as compared to the control group (0.48 to 1.79) due to an increase in oral hygiene compliance among participants of the study group. Scheerman JFM et al. (2019) showed a decrease in plaque and gingivitis with increased fluoride use [23]. McNab M et al. (2016) quoted that the use of educational video instigates the participants to use less sugar in their day-to-day lives [24]. Free C et al. (2015) [25] and Toniazzo MP et al. (2019) [26] both showed improved self-reporting and disease management with less amount of plaque and gingivitis with the help of using mobile applications and SMS reminders. Jadhav HC et al. (2015) used SMS reminders and found a statistically significant decrease in the mean oral hygiene index (OHI) (3.79 to 2.88) and gingival index (GI) (0.31 to 0.16) among the study group [20]. Bankhole et al. (2013) showed that the oral hygiene of the participants improved significantly by 28.6% among those who watched oral health education videos [27]. The help of follow-ups and retention rates showed that there was an increase in care and continuity of services among five studies.

The systematic review conducted by Free C et al. (2013) also signified the positive health change behavior with the help of mobile-based intervention [25]. This review considered 75 studies and found that regular text messaging increased adherence to smoking cessation habits and anti-retroviral therapy. Similar study results were observed by Toniazzo MP et al. (2019) who did a systematic review considering 15 research articles with a 1402 sample population [26]. The study result was in favor of positive reinforcement with the help of text messaging and mobile applications on oral hygiene maintenance and a decrease in gingivitis and plaque levels. Bassi A et al. (2018) conducted systematic research on 318 articles with 125 primary research and 193 review papers in India with the objective of assessing the effectiveness of the role of MHealth interventions in strengthening the Indian Health System [21]. He concluded by stating that MHealth initiatives have proved beneficial in strengthening the health care delivery system in India.

Among these, six studies did a chi-square analysis, three used the ANOVA test and four did a T-test analysis. Apart from the Wilcoxon Mann-Whitney analysis [19,22], X2, and I2 tests [25,26], multiple logistic regressions [27] were also used to do a statistical analysis. Self-reporting bias [17], follow-up, and social desirability bias [24] were also mentioned in the studies. Out of all the selected studies, nine have shown a statistically significant increase in oral health literacy and oral hygiene practices during the intervention and follow-ups. All the studies stated improvement in oral health by using digital media except one study conducted by Al Bardaweel et al. (2018) [28], in which traditional learning through educational pamphlets was used. This study was conducted among primary school children who were not very well familiar with digital technologies.

Discussions

The impact of digital media is very strong and that benefit can be utilized to create positive reinforcement in the population about their oral health regardless of their age. The revolutionary development of digital media in day-to-day life and its reach to the community is the window of opportunity for influencing oral health behaviors through oral health education, social marketing, and oral health promotion interventions. As far as developing nations like India are concerned, this technology can help in achieving our goals of Universal Health Coverage in the most economical way possible. It has enhanced information sharing across the world, giving people much greater access to facts, figures, statistics, and similar, allowing that information to circulate much faster [32]. The paradigm shift involves symbiotic networks of smart medical devices, smartphones, or mobile personal computing and communication devices. This reinforces experimental learning, which is the process of learning by doing. Various applications have been used for oral health education and promotional activities, such as toothbrush timers, tips for better oral hygiene, oral hygiene alert and reminders, oral health educational content, tracking of oral health behaviors, tracking of dental appointments, etc. [33]. These applications provide oral health-related information and simultaneously engage people to follow oral hygiene habits leading to actionable outcomes and improved oral health. A few among these are BrushDJ, MyDentist, Text2floss, MyFitStrip, ToothSavers, etc. [34,35]. It has also been used in a range of health contexts, including chronic disease [36], winter preparedness [37], general school health [38], physical activity [39], substance abuse [40], mental illness [40,41], dietary behaviour change [42,43], and breastfeeding [44]. It has also been evaluated for efficacy in enabling health-promoting capacity through processes such as information seeking [45] and holistic self-participatory care [46]. Further, these media also have been used in specific disease contexts such as obesity [47] and breast cancer [48]. This systematic review evaluates the effectiveness of digital media in oral health promotion. The findings reveal that there is a wide variety of interventions addressing populations of any age. This diversity broadens the horizon of oral health services, which significantly can aid in reducing the oral health burden on society. Due to the rapid rise in mobile phone usage over the past decade, digital media has become a popular platform for health-related behavior modifications [49].

This review has shown that digital media can increase the early reporting of dental diseases [24], increase oral health knowledge, attitude, and practices [18,22,23], and positive behavior change toward oral health practices [20] with diet modifications [23]. Using various digitalized interventions led to an increase in the oral hygiene status of the participants with a significant decrease in the scores of various indices like plaque index, calculus index, or gingival index. The interactive session and active participation by the study subjects have significantly raised the level of compliance and retention with the therapy provided. This has created a huge impact on preventive practices and continuity of care among the participants [19,25]. Tailor-made, customized interventional approaches using interactive methods during the discussions and reinforcement using text messages had minimize oral health literacy barriers and improved parental cognitions with respect to their child’s oral health [17]. Digital media like videos and text messages and mobile applications were found to be instigating both immediate and sustained self-reported behavior change [23] thus causing a gigantic impact on cognitive behavior. This cognitive behavior change may enable lifestyle modifications like quitting tobacco, decreasing sugar consumption, increasing oral hygiene compliance, and using fluoride therapy [20-24] Though the comparatively short follow-up period may not be sufficient enough to provide a quantifiable change in complex behavior of an individual but according to Wakefield et al., behavior change may also occur indirectly because health messages can set an agenda [50]. This increases discussion about a particular health issue within an individual’s social environment, which, in combination with individual exposure to the message, may reinforce (or undermine) a specific change in behavior [50].

MHealth strategies have been used in medical fields also. Mobile applications have been used in the self-management of diabetes that can be considered as an adjunctive intervention to standard treatment protocol [51]. Systematic and meta-analyses have been done to review the effectiveness of digital media in adherence to medical therapy, along with the maintenance of blood pressure, healthy diet, and exercises [52,53]. The same results were shown by the Itteffaq Muhammad et al. (2018) and Asfaw Aatnafu et al. studies [54,55]. The review has considered the broad age group which helps in the generalizability of the results and strongly recommends the use of this opportunity to build relationships with existing and future patients. Continuous reinforcement via these techniques helps the patients to adopt healthy behavior and may decrease the incidence of oral diseases.

Limitations

Only the English language was considered in this review. Studies conducted before 2010 and those reporting oral health schemes to provide insurance benefits were not reviewed.

Recommendations

Although digital media has the capacity to improve efficiencies and coverage, the technology itself does not guarantee success. High-quality, evidence-based interventions that demand individual participation are crucial to success. An important challenge for public health is the rapidity of change that may outpace the currency of evaluation and publication, creating a space for many interventions to flourish without solid evidence and for effective interventions to lose relevance. Such challenges compound the need for ongoing and timely research to monitor and evaluate these new trends while not losing sight of the general evidence-based principles that underlie all effective health promotion.

## Conclusions

This review mainly focuses on oral health education, knowledge, attitude, and practice toward the oral health behavior of patients. From the results observed, it can be concluded that digital media usage in today's life can help us to enhance oral health literacy, improve oral health, and thus play a pivotal role in achieving overall health.

## Appendices

**Table 5:** Summary of the Studies Included in This Review K - Knowledge A - Attitude P - Practice OHI - Oral Hygiene Index GI - Gingival Index OHIS - Oral Hygiene Index Simplified

S. No	Author	Country/ Study Design	Study Sample	Intervention	Comparison	Outcome
1	Scheerman JFM et al. 2019 [23]	Netherland/ RCT	132 Adults Undergoing Orthodontic t/t	White Teeth App	Usual Care	Decrease plaque, gingivitis, fluoride use, KAP 20.08%-22.50%
2	Al Bardaweel S et al. 2018 [28]	Syria/ RCT	220 1^o^ School Children	e- Learning	Leaflet Learning	KAP leaflet (54.94 to 89.12) e-learning (55.50 to 74.66)
3	Gholami et al. 2017 [18]	Iran/ RCT	543 Adults age 18-50 yrs.	Animated clips on oral health on national TV for 10 days	Before Intervention	KAP 0.29 to 0.61
4	Zotti F et al. 2016 [22]	Italy/ RCT	80 Adolescents Orthodontic t/t	Whatsapp Messages	Usual Instruction	Improving oral hygiene compliance. Decrease in PI- SG 0.41 TO 1.06, CG 0.48 TO 1.79.
5	Makvandi Z et al. 2015 [17]	Iran/ RCT	90 Mothers of 1-2 yrs Old Children	Whatsapp Messages	No Information	K 4.80 TO 6.20, A 77.62 TO 95, P 58.29 TO 83.35
6	Harish C Jadhav et al. 2015 [21]	India/ RCT	400 College Students	SMS	No Information	Mean OHI (3.79 to 2.88) GI (0.31 TO 0.16) study group
7	Bankhole Olubunmi et al. 2013 [27]	Nigeria/ RCT	Primary School Children	Educational Video	Verbal Dental Health Education & No Information in Control	Decrease of OHIS score video group 28.6%, verbal group 23.4%, control group 14.1%
8	Fatemeh Mohamadkhah et al. 2013 [19]	Iran/ Quasi Experimental	300 (10-12 yrs. old) Girls	Films/ Lectures	No Information	K (1.93 TO 2) A (1.86 TO 2.05) P (2.07 to 2.02)
9	Mcnab M, Skapetis et al. 2016 [24]	Sydney/Non- Randomized Trial	253 General Population	Oral Health Educational Video	Before Intervention	Decrease sugar consumption, KAP (57.5% to 78.2%)
10	Toniazzo MP et al. 2019 [26]	Brazil/ Systematic Review	1402 Adults, mothers of young children, Adolescents	Mobile Application (app) and SMS	Usual Oral Hygiene Instruction	Decrease plaque and gingivitis
11	Caroline Free et al. 2015 [25]	London/ Systematic Review	Health Care Consumers	Mobile App	Face-to-Face Counseling	More cases of self-reporting, improvement in disease management
12	Abhinav Bassi et al. 2018 [20]	India/Systematic Review	Health Care Consumers	Mobile-Based Health Intervention (Telemedicine/MHealth)	No Digital Intervention	Improvement in education level and positive behavior change
